# A New eHealth Investment Appraisal Framework for Africa: Validation

**DOI:** 10.3390/ijerph20146426

**Published:** 2023-07-21

**Authors:** Sean C. Broomhead, Maurice Mars, Richard E. Scott

**Affiliations:** 1 Department of TeleHealth, School of Nursing & Public Health, College of Health Sciences, University of KwaZulu-Natal, Durban 4041, South Africa; mars@ukzn.ac.za (M.M.); ntc.ehealthconsulting@gmail.com (R.E.S.); 2 Health Information Systems Program South Africa, Pretoria 0181, South Africa; 3 African Centre for eHealth Excellence, Cape Town 7130, South Africa; 4 Department of Community Health Sciences, Cumming School of Medicine, University of Calgary, Alberta, AB T2N 1N4, Canada

**Keywords:** eHealth, digital health, investment appraisal, economic appraisal, Africa, low-income countries, middle-income countries

## Abstract

(1) Background: Decisions to use eHealth are complex and involve addressing a large opportunity cost. Sound choices are essential. Weighing up investment options is challenging in resource-constrained settings where there are frequently insufficient economics data and expertise to conduct adequate appraisals. To address this, a new eHealth Investment Appraisal Framework (eHIAF) for Africa has been designed and developed. The aim of this paper was to validate the new framework to consider whether it is fit for purpose and to refine it as needed. (2) Methods: An online survey of purposively selected eHealth experts was used to conduct a desktop validation of the proposed eHIAF for Africa. The survey covered the framework development process, structure, content, completeness, and utility. Expert opinions were charted, and a reflective and iterative process used to assess the tool and extract recommendations for refinement. (3) Results: Eleven eHealth experts who completed the survey had experience in African countries and elsewhere. The majority agreed with the eHIAF for Africa development approach and output. They provided valuable suggestions for minor refinements and felt that with these amendments, the eHIAF for Africa would be ‘fit for purpose’. (4) Conclusions: The eHIAF for Africa is considered appropriate for use by policy- and decision-makers working in resource-constrained settings who face the task of selecting optimal eHealth investments. It has the potential for applicability beyond Africa and the framework should now be tested in African countries.

## 1. Introduction

Decisions to use eHealth (Information and Communication Technologies (ICTs) for health) are complex [1,2]. The implementations are high risk, extend across several years, and affect several stakeholder types including patients, healthcare workers, healthcare organisations, insurance companies, and governments [3]. Since opportunity costs for these investments “can be large, countries must make choices about which interventions to scale up” [4]. Informed, clear investment decisions that achieve long-term sustainability are essential. Nevertheless, economic evaluation methods that help decision-makers to articulate eHealth’s relative value for money (VFM) are often inadequate to determine whether the eHealth initiatives will strengthen healthcare, rather than weaken it further [5].

Africa lags behind other regions for most health indicators and this trend is forecast to continue [6,7,8]. In response to these challenges, the African Union launched ‘Agenda 2063: The Africa we want’ [9] with the aim to establish a high standard of living associated with good health and well-being. However, Africa remains hampered by a significant disease burden and insufficient expenditure on healthcare [8,10,11,12]. African Union countries signed the Abuja Declaration in 2001 and committed to spending 15% of their annual budget on health sector improvements, but the average spend achieved is only 7.2% [8,12]. The annual health expenditure per capita across sub-Saharan Africa in 2018 was USD 83.25, less than one-tenth of the global average of USD 1111.08 [13,14]. Furthermore, countries in the WHO African region spend only 7% of their health expenditure on infrastructure (which includes ICT), significantly below the recommended 33% [13]. Improvements to financing and infrastructure have been recognised as necessary for African countries to achieve UHC [15].

Under these circumstances of significant disease burdens, but with constrained budgets with which to address them, the use of any portion of the health budget for something new requires sufficient and evidence-based motivation.

The WHO promotes a drive towards ‘Universal Health Coverage’, defined as “…all people have access to the full range of quality health services they need, when and where they need them, without financial hardship” [16]. To advance UHC, the WHO World Health Assembly and WHO Regional Committees encourage member states to embrace opportunities to use eHealth for strengthening health systems [17,18,19,20,21]. During the COVID-19 pandemic, there was an increase in the promotion and uptake of eHealth [19,22]. Digital tools were implemented for remote consultation, contact tracing, and patient management to support the COVID-19 response [23,24,25,26,27].

The WHO Regional Office for Africa encourages African countries to use eHealth to help respond to high disease burdens despite constrained resources [28,29], and there is growing recognition of the role of eHealth in attaining UHC in Africa [30,31]. This is likely to be supported by new generations of African health workers who use technology resources frequently and have positive attitudes towards eHealth [32]. Nevertheless, in resource-constrained African countries, which need UHC the most, the barriers to using eHealth including finance and infrastructure barriers are significant [30,33]. eHealth is not possible without adequate infrastructure and connectivity [34,35], and the digital divide increases their costs.

Competition for resources occurs in most countries and decision-makers need to confirm the socio-economic benefits of the possible options. Under the constrained conditions described above, a robust investment case is especially important to clarify the value of eHealth when competing with other investment options. The need to appraise eHealth investments has been recognised for more than a decade [36,37,38].

In resource-constrained settings, where economic data and expertise may be limited, it is particularly difficult to determine whether a proposed eHealth initiative provides the best investment opportunity to strengthen healthcare [39,40]. The current practice of economic appraisal of eHealth investments is not adequate in African countries [41,42,43]. Economic appraisal tools are also inadequate [5], and there are substantial gaps in the digital health economic appraisal literature globally, and in studies from African countries in particular [4]. Worse still, most African eHealth initiatives do not have a prior assessment of any kind, let alone economic appraisal [43,44,45,46].

eHealth investment appraisal provides “a process to evaluate which information and communication technology investment in health produces optimal net benefits” [47]. This planning process seeks the most advantageous balance between VFM and affordability while maintaining strategic alignment [48]. It combines economic evaluation, which provides a comparative analysis of the costs and consequences of one or more interventions [49], with other perspectives essential for successful eHealth implementation.

The World Health Organisation has noted that “To realise their potential, digital health initiatives must be part of the wider health needs and the digital health ecosystem and guided by a robust strategy that integrates leadership, financial, organisational, human and technological resources and is used as the basis for a costed action plan which enables coordination among multiple stakeholders” [50]. It is essential to ensure accountability for eHealth investment decisions, particularly in low- and middle-income countries where eHealth investments compete with other healthcare needs for scarce resources, and where the implications of failed initiatives are magnified. Therefore, a robust investment appraisal of each option is needed for a satisfactory, affordable, and sustainable action plan.

To address this need, a new eHealth Investment Appraisal Framework (eHIAF) for Africa has been developed, informed by the literature [51]. It is based on the Five Case Model (FCM) and is designed for settings that lack sufficient economics expertise and data. The proposed new eHIAF for Africa has six stages: establish a compact with key stakeholders, collect data, generate an economic model, establish affordability metrics, iterate to consider options and identify optimal investment choices, and establish a sustainable implementation. Each stage addresses one or more of 23 attributes. The framework structure and the 23 attributes are summarised in Table A1 in the Appendix A.

Those making investment decisions in African countries frequently lack the economics data and/or expertise to perform adequate eHealth investment appraisals [39,40]. Therefore, an appropriate eHIAF for Africa must be accessible to them [47] and remain useful as their access to economics data and expertise grows. The aim of this paper was to validate the new framework through a survey of international digital health experts from Africa and elsewhere and consider any refinements they proposed.

## 2. Methods

Conceptual validation of the eHIAF was achieved by combining findings from a prior literature review with expert opinion as an effective validation approach [52]. The goal was to determine if the tool was ‘fit for purpose’, in other words, “capable of consistently guiding the process it is supposed to and meeting the operational needs of its intended users” [53]. A literature review has been reported [47,51], and the expert opinion is reported below. Gaining expert opinion involved the use of an online survey of purposively selected digital health experts. Selection was based on their active involvement in digital health leadership and implementation, as demonstrated by the length of their time working in digital health and their contributions to conferences, publications, and reports. Experts were selected from three geographic and economic classifications: low-income countries (LICs) and middle-income countries (MICs) in sub-Saharan Africa; LICs and MICs outside Africa; and high-income countries (HICs). Invitations were issued until there were four acceptances for each group. Expert selection and sample size were based on a previously published approach [53].

Selected experts were sent an initial introductory email inviting participation. Those who accepted the invitation were sent a more detailed email including a written consent form for signing, a pre-publication copy of a paper describing the eHIAF for Africa and its development process [51], and a link to the self-administered, anonymous, online survey on Google Forms. The consent form clarified the participants’ rights and obligations. After providing written informed consent, each participant accessed the survey and was given two weeks to complete it. The survey was administered online between December 2022 and February 2023.

The survey covered the following domains relating to the eHIAF for Africa: development process, structure, content, completeness, and utility. It was made up of 48 compulsory closed-ended questions (37 Likert scale, nine multiple choice and two dichotomous) with 40 opportunities to provide optional open-ended clarification or explanation through free-text responses. A five-point Likert scale was used (5 = agree entirely, 4 = mostly agree, 3 = unsure, 2 = mostly disagree, 1 = disagree entirely). The optional free-text responses provided opportunities for diverse and “authentic” contributions in “an unaided freeform way… to share details about their experiences that the researcher did not anticipate” [54]. The survey tool was developed by one author (SCB) and tested extensively with the other authors (MM, RES). Iterative refinements were made to the questions and structure until the tool was regarded as user-friendly and unambiguous. The questions are listed in Table A2 in the Appendix A and are summarised in Table 1.

Responses were collated in an Excel spreadsheet (Microsoft 365), and Likert responses were colour coded. The overall pattern of responses was viewed for trends, and the findings were compared both vertically (all responses to one question) and horizontally (one respondent’s answers to multiple questions) to facilitate identifying patterns in the data. Responses to Likert-type questions were further aggregated into three categories, ‘agree’ (scores 4 or 5), ‘disagree’ (scores 1 or 2), or ‘uncertain’ (score of 3), and the median scores were recorded for each question. Open-ended responses were collated within these categories and examined for further insights and/or recommendations for strengthening the framework.

Primary review and inductive analysis of the charted data were conducted by one author (SCB). Thereafter, a reflective and iterative process was followed independently and collectively by all authors to reassess the data, address and resolve inconsistencies by consensus, and identify changes that might strengthen the framework. Where the authors reached a consensus regarding a change proposed by the respondents, the eHIAF for Africa was refined accordingly. Reporting employed a narrative approach guided by the Standards for Reporting Qualitative Research [55]. Tables and a figure were used to visualise the responses and to present the refined eHIAF for Africa.

Ethics approval for the survey was obtained from the UKZN Humanities and Social Sciences Research Ethics Committee (HSSREC/00004976/2022), and the respondents provided written consent to participate.

## 3. Results

### 3.1. Selection and Demographics of Digital Health Experts

Of the 20 experts invited, twelve accepted the invitation, and eleven ultimately completed the anonymous online survey tool in full. Respondents A, B, C, and D were invited from Sub-Saharan Africa, respondents E, F, and G from MICs outside Africa, and H, I, J, and K were from HICs. Countries they had worked in most were various combinations of LICs, lower middle-income countries (LMICs), and upper middle-income countries (UMICs) (World Bank definitions [56]) both within and outside Africa (Table 2). None reported having worked most in HIC environments.

Six respondents had used either cost–benefit analysis (Experts B, D, I, K), cost-effectiveness analysis (F), or both (G). Four respondents had not used either (A, C, E, H), and one was unsure about these economic methods (J). Nine respondents felt that there were insufficient economics data available for LICs and MICs to conduct eHealth investment appraisals (A, B, C, E, F, G, H, I, K). One respondent (D) felt that sufficient data were available and referred to examples of available macro data. Another (J) was unsure and suggested that the private sector might have access to relevant data. The pattern of answers was similar regarding the availability of economics expertise, with six answering that sufficient expertise was not available (A, F, G, H, I, K), and two that it was available (D, E), commenting that academic institutions and consulting firms had this expertise. Three (B, C, J) were unsure and suggested that there may be expertise available that had not yet been used for this type of work. Simple observation of the colour coded data showed that the responses of those who had experience working with economic appraisal methods were similar to the views of the other experts.

### 3.2. Respondents’ Impressions of the New Framework

#### 3.2.1. Overall Impressions

The respondents’ answers to closed-ended questions about the new framework showed majority agreement (353 out of 385; 91%) with most respondents (eight out of eleven) agreeing with all aspects of the new framework (Table 3). The respondents’ answers were supported with additional comments, with 132 of the 440 optional open-ended responses (30%) being completed. Nearly half of the comments (*n* = 63) provided explanations for the answer to Likert scale questions, and the others (*n* = 69) provided suggestions for strengthening the eHIAF for Africa. Simple observation showed that those who had worked most in African countries expressed a similar agreement with the new framework to the other experts.

#### 3.2.2. Developmental Process and Structure

Regarding the development process, nine respondents agreed with the progression of the four steps, one was unsure (H), and one disagreed (A), commenting that there should be a preceding step “*determining the need for the framework*”. Most respondents found the individual steps to be appropriate, with nine agreeing with the first step, 11 with each of the second and third steps, and 10 with the fourth step. Those who did not agree chose ‘unsure’, and none disagreed. Most respondents (nine) found the structure easy to understand, and one chose ‘mostly disagree’, suggesting that “it would be helpful if the attributes had names and then the questions were like the assessment question to assess the attribute” (H). One chose ‘unsure’ (A) without clarifying the answer. Ten found that the structure was helped by the alignment of each appraisal attribute with the FCM cases and with the eHIAF stages, and one chose ‘mostly disagree’ (A) without clarifying the answer. Eight respondents agreed with the grouping of attributes into six stages, and the remaining three respondents chose ‘unsure’. One respondent suggested that the presentation of the structure could be strengthened with “*a flowchart graphic*” (K).

#### 3.2.3. Content

Most respondents (10 out of 11) expressed agreement with the 26 questions about the framework content (264 out of 286 answers; 92%). Disagreement was only noted on four occasions (1%), and in each of these, only one respondent disagreed (H) and the selection was ‘mostly disagree’. A subset of 23 questions within the content section specifically addressed the eHIAF attributes. For these questions (Table 4), similar results were noted (235 of 253 answers; 93%) and the median scores for the individual attribute questions were all either four or five out of five, indicating general agreement.

All eleven respondents found the attributes easy to understand. Each respondent proposed at least one refinement such as considering a “*Delphi survey to identify potential attributes from unpublished sources and grey literature*” (C) and expanding on “*how to reinforce the importance of comprehensive and adequate options analysis*” (K). There were also comments suggesting that the keywords used in attributes be defined. These included the meaning of ‘adequate’ in attribute 3 (Experts J and K), the ‘all’ of ‘all issues of concern to users’ in attribute 4 (A, C, H, K), ‘generalisable’ in attribute 5 (B, C, E), ‘relevant’ in attribute 7 (E), ‘appropriate’ in attribute 8 (Expert E), ‘discount rate’ in attribute 10 (J), what ‘analysis of options’ means in Attribute 11 (C, H), ‘consequences’ in attribute 12 (C), ‘risk’ and ‘optimism bias’ in attribute 13 (H), and ‘practical plan’ in attribute 18 (A). Regarding attribute 6, ‘Has an appropriate timescale been set?’, a suggestion was made to reinforce the need for continuous investment throughout the lifetime of a project and for large projects such as EMRs to have timescales of decades rather than years (J).

Other recommendations included the extension of “*amortisation over the life-span of the digital health intervention*” when dealing with costs and outcomes (K), considering extending clinical effectiveness “*beyond clinical to include public health benefit*” (C) and expanding the question on partnerships to “*include all relevant sectors*” (C). There were suggestions that attribute 4 (‘Does the appraisal include all issues of concern to users?’) might work better if ’all’ was changed (H). One respondent proposed that it be changed to ’most’ (C). Two respondents felt that attribute 18 (‘Is there a practical plan for delivery?’) and attribute 19 (‘Are clear delivery milestones provided?’) were closely related and could be linked (A, G).

One respondent (H) disagreed with three attributes and provided comments. First, they proposed further clarification about whether the ‘costs and outcomes’ in the attribute ‘Are all important and relevant costs and outcomes for each alternative identified?’ were for the eHealth appraisal or the implementation. Second, they confirmed agreement with ‘Were sensitivity analyses conducted to investigate uncertainty in estimates of cost or consequences?,’ though raised concern that there would be insufficient data available to conduct the analyses. Third, they expressed concern that the definition provided for ‘Is there a plan for building partnerships?’ was inadequate.

Comments that followed the minority of ‘unsure’ answers (24 out of 385; 6%) included several of the requests for keyword definitions identified above, plus one question about how the framework’s ‘robustness’ would be tested (A). Respondent H requested further explanation of why the two published checklists were chosen as the foundation “*to make it clear that these are attributes of economic appraisal and you were using the other frameworks from the health sector to augment this list and adapt to the eHealth* [context]”. The respondent suggested that a user guide would be useful.

#### 3.2.4. Completeness

Most respondents (10 out of 11) agreed that the eHIAF for Africa ‘addresses all important eHealth investment appraisal issues’. Only one (E) disagreed and suggested that the framework should “*deal directly with governance*”. Further comments were to include “*a governance structure*” in stakeholder engagement (J), refine the framework iteratively “*during implementation of the framework*” to “*define a maturity framework*”, and “*describe tools and approaches for implementing the framework*” (C). Respondents suggested that consideration be given regarding how to include new developments such as “*AI, Internet of Things, confidentiality, and security*” (D), and to address “*compliance with local laws and regulations*” including adding pre-appraisal questions “*to ensure time is not wasted on an economic appraisal on a non-viable initiative*” (H). Regarding resourcing, respondents suggested that the framework “*call out human resources as it does connectivity*” (H) and require “*a clear human resource plan*” (J).

Two new attributes were proposed by respondent J: “*Is there a data management plan?*” to include issues such as security, privacy and consent and “*Have ethical and equity issues been considered and planned for?*”. A suggestion was made to include “*political economy*” in the attribute dealing with change management (F) and to consider “*re-usability*”, recognising that some outputs can “*enable or contribute to solutions for other sectors*” (I). Respondent C suggested that procurement plans should include transition to a “*sustainability model*” and that sustainability could be separated into a “*a group of its own… due to its importance*”.

Four respondents suggested that the eHIAF for Africa should be more inclusive of infrastructure. Several suggestions were provided to extend the attribute ‘Is there adequate connectivity?’ such as “*to include ‘IT infrastructure’ rather than singling out connectivity*” (C), to address “*digital infrastructure more broadly (not connectivity alone)*” (I), and to include issues such as “*infrastructure availability*” (A). A further comment (K) was that the attribute should be expanded to include “*power availability and hosting services*” and “*the policy enabling context—including data security, privacy, confidentiality, sharing, and exchange*” to create “*an adequate ICT enabling environment*”. Respondent K also proposed adding an attribute or expanding the existing attribute to read “*Is there an adequate ICT enabling environment?*” and asked how the eHIAF for Africa could incorporate enterprise architecture that “*a lot of countries in Africa and Asia [are] committing to*”.

#### 3.2.5. Utility

Although all respondents (*n* = 11) indicated that they would use the eHIAF for Africa to appraise future eHealth initiatives, most (*n* = 8) further clarified that their use of the eHIAF would require that it be strengthened based on the feedback they had provided. Similarly, most (*n* = 10) indicated that they would also use the eHIAF for Africa to appraise existing initiatives, with most (*n* = 8) clarifying that their use of it would be dependent on it being strengthened with their feedback. A comment about the importance of the framework being practical and usable was “*Our sector tends to produce unwieldy, 100+ page guidance documents that no one ever reads, so having a simple presentation of a framework, which can be backed up by more detailed guidance on how to evaluate each attribute, is helpful*” (J).

One respondent (J) raised four considerations. First, “*How do you score the answers to each question?*”, suggesting “*a scale of 1 to 10 because not all questions will have a clear yes/no answer*”. Second, suggesting “*a how-to guide towards coming up with the final ‘go/no-go’ decision* [whether to invest or not invest]”. Third, that there would be value in clarifying “*who the intended user of the tool is*”, and fourth, that the tool could be used “*to empower governments*” to assert their agenda with donors.

#### 3.2.6. Applicability

All eleven experts agreed that ‘If applied effectively, the eHIAF (including any revisions proposed) will help to identify optimal eHealth initiatives to be prioritised’. The broader applicability of the framework was noted with the comments “*the eHIAF is not just applicable to Africa… low resource settings striving for digital transformation have a lot more in common than not*” (K), “*I think it can be reiterated more strongly throughout that the eHIAF is not just applicable to Africa*” (K), and “*connectivity may be the only question that makes this more relevant to Africa/LMICs otherwise this is applicable to all contexts*” (J). Regarding the re-usability of outputs, a respondent suggested, “*It is important to recognise that certain outputs delivered as part of an eHealth initiative can also enable or contribute to solutions for other sectors. For instance, digital registries or preventive health communication solutions can be easily re-used for other health applications or even cross-sectoral, e.g., e-learning*” (I).

#### 3.2.7. Alternatives

Three respondents highlighted similarities with other approaches: “*This is similar to the TOGAF approach of defining the current state (step 2), defining the target state (step 1) and crafting a sequence plan (step 3–4)*” (E); “*Transform Health has recently published a Conceptual Framework to guide investments and action towards health for all in the digital age*” (G); “*Not sure if the eHIAF is a ‘new’ framework per se, or an enhancement of the DHIF or the consolidation or extension of the FCM*”, suggesting that the “*work of the DHIF using the LiST and addressing morbidity avoided and lives saved estimates*” to help decision-makers to “*equate digital health investments with other costs like diagnostics, therapeutics and medicines*” (K).

### 3.3. Framework Amendments

The respondents’ comments were used to refine and strengthen the eHIAF for Africa. Amendments were aimed at improving clarity and reducing complexity, and in the case of uncertainty or a lack of consensus, the original wording was retained. All of the refinements are listed in the two tables in the Appendix A. Table A3 in the Appendix A lists how the respondent comments were applied to the eHIAF. Table A4 in Appendix A provides a supporting narrative to guide users to apply the framework attributes.

## 4. Discussion

The majority (eight) of the eleven eHealth experts agreed with all aspects of the eHIAF development approach and its structure, content, completeness, and utility. Of the possible 385 responses to 35 Likert questions, 92% were positive (agreement), with only 6% of negative responses (disagreement) across eight questions. While the eHIAF was designed for Africa, two respondents (J, K) felt that it was suitable to be used more widely. All of the respondents provided valuable suggestions for minor refinements, related to the language, definitions, and scope of the attributes that after adoption provided a final refined eHIAF. In addition, the respondents’ suggestions resulted in a more detailed narrative table (Appendix A Table A4) that provides the necessary guidance for the application of the framework attributes.

The respondents’ profiles covered a geographic and organisational spread likely to represent a range of perspectives and insights that would promote meaningful responses to the survey. Of the 11 respondents, the majority had experience working in Africa (eight) and had worked for implementing organisations (seven). The majority of respondents also agreed that the economics data (nine) and expertise (six) needed to conduct eHealth investment appraisals were limited in LICs and MICs. The few respondents who were unsure or disagreed referred to the availability of macro data, rather than the granular data about the costs and consequences needed to appraise an eHealth business case. One respondent (J) suggested that private sector resources could be leveraged to address economics data and expertise shortages. This would support the full application of the eHIAF step addressing the economic case. Another respondent (E) suggested that expertise might be available through academia. The potential for these additional specialist health economics resources to complement the limited available economics resources of African countries has been recognised, though is in short supply [47].

In relation to framework refinements, all respondents found the attributes easy to understand. Their proposed changes served to reinforce the scope and purpose, clarify the language, and strengthen the definitions. In applying the refinements, careful consideration was given to the respondents’ comments about the importance of the framework being practical and usable, and for there to be a simple presentation of the framework, which can be supported by more detailed and complex guides as necessary.

Regarding the four occasions of disagreement with an attribute, all were from one respondent (H) and the respondents’ comments were used to guide the eHIAF refinements. One disagreement related to attribute 21, ‘Is there adequate connectivity?’, which was revised to read ‘Is there an adequate ICT enabling environment?’. This emphasised the inclusion of broader ICT infrastructure elements and addressed comments provided by several other respondents (A, C, I, K). Two disagreements were addressed by editing the definitions of attributes to clarify that ‘costs and outcomes’ refer to the eHealth intervention and not to the appraisal, and to provide a clearer explanation of what ‘partnerships’ entails in attribute 22. The fourth disagreement raised concern that African countries might have insufficient data to conduct sensitivity analyses. This reinforced the need for a framework that is applied flexibly, as in the proposed eHIAF for Africa.

**Figure 1:**
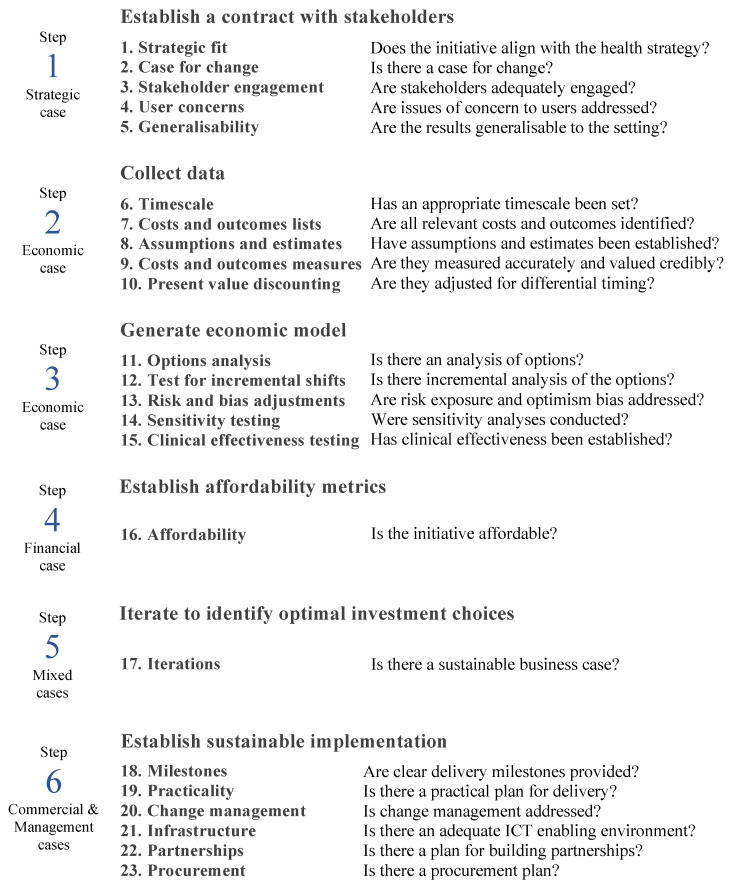
The eHIAF for Africa flowchart.

Alignment of each appraisal attribute with the FCM cases and with the eHIAF stages was maintained and a flowchart was added as requested by Expert K (Figure 1). Potential redundancy between the flowchart and a supporting narrative table were resolved by simplifying the figure and enhancing the narrative to provide more detailed guidance in the utilisation of the attributes (Table A4, Appendix A). The suggestion to consider the re-usability of outputs (I) was added to the attributes dealing with the costs and consequences so that both the cost-sharing and wider benefits realisation opportunities could be explored.

### 4.1. Respondent’s Suggestion Not Implemented

Only one suggestion, while having been considered carefully, may be regarded as having not been implemented. The respondent (D) proposed an exploration of how to include new developments such as “*AI, Internet of Things*”. The artificial intelligence (AI) aspect of this topic was addressed in the recent publication of a digital health economic appraisal approach [4]. The extent to which emerging technologies like AI will affect investment appraisal methods is not yet clear and has therefore not resulted in any amendment to the eHIAF for Africa. Nevertheless, the existing eHIAF for Africa can be used for the appraisal of an AI digital health initiative and would proceed as follows. Attributes 1 to 5 would clarify the initiative’s intentions and whether it is fit for purpose, attributes 6 to 17 would help to further clarify the value for money of the intended proposition, and attributes 18 to 23 would demonstrate the ability to implement effectively and sustainably. Together, these would provide an investment case that would assist African decision-makers. These could be updated as future evidence appears that describes any unique additional requirements that need to be met for the investment appraisal of emerging digital health technologies such as AI.

eHealth is complex and there are dynamic interrelationships between its stakeholders and evolving technologies. Insights about how best to implement the eHIAF for Africa may be derived from further investigation using in-depth qualitative data collection techniques such as focus group discussions.

### 4.2. Relationships with Other Frameworks

A recent systematic review noted that a key challenge in assessing digital health value is the complexity of evaluating clinical, organisational, and economic aspects simultaneously [57]. Several respondent comments reported in the results reinforce the review’s five recommendations of what should be considered in the measurement of the value of digital health initiatives, which are all addressed in the eHIAF for Africa (Table 5).

Three experts commented on possible relationships with other frameworks: The Open Group Architecture Framework (TOGAF) (E), the Conceptual Framework published by Transform Health (G), the Digital Health Impact Framework (DHIF), and FCM (K). TOGAF is commonly used as a reference framework for describing enterprise architecture and has been used to design health information systems [58,59]. TOGAF’s unique approach to generating ‘principle statements’ may provide a useful method for recording the users’ needs and clarifying the desired health outcomes. However, while TOGAF is an approach for architecture design, the eHIAF addresses the unique requirements of eHealth business case investment appraisal. These roles are potentially complementary, but do not replace one another. The related request by one respondent (K) to consider how to incorporate enterprise architecture (EA) into the eHIAF was not addressed for similar reasons. EA describes the systems architecture of a whole enterprise [60], and while high-level alignment between organisational architectures and frameworks makes sense, the EA role is different to the business case investment appraisal role of the eHIAF for Africa.

Further, the conceptual framework published by Transform Health [61] prioritises nine investment areas and its macro-level costings provide a high-level costing guide for countries. These do not assist officials in selecting the optimal implementation approaches when faced with competing options and the need to manage the related opportunity costs. The relationships between eHIAF, the DHIF, and the FCM have been described in this paper and elsewhere [47,62]. While both the eHIAF and the DHIF are based on the FCM, the structure of the eHIAF has significant additions to the strategic, management, and commercial cases that are different to the DHIF. The eHIAF additions are unique to the circumstances experienced in African countries. The eHIAF for Africa is an expansion of the DHIF, and the DHIF may provide relevant material for those who implement the eHIAF.

In addition, the eHIAF for Africa has similarities with the Framework for Economic Evaluation of Digital Health Interventions (FEEDHI) published recently (2023) by the World Bank Group. The FEEDHI is mentioned due to its unique approach to the evaluation of AI initiatives. The World Bank framework ‘aims to assist in generating economic evidence to improve health in a digital world rather than viewing DHIs as isolated health system investments’ [4]. It emphasises that ‘methodological transparency’ will help to improve ‘the overall usefulness of economic evaluations of digital health interventions’ [4,63]. There are similarities between the five steps of the FEEDHI and the eHIAF for Africa. The first step of the FEEDHI to ‘determine the context’ aligns with the step one attribute of the eHIAF for Africa, ‘Establish a contract with stakeholders’. The last two steps of the FEEDHI ‘set the analytical principles’ and ‘represent the value proposition’ align with steps 3 to 5 of the eHIAF, which are ‘generate economic model’, ‘establish affordability metrics’, and ‘iterate to identify optimal investment choices’. Two distinguishing features of the World Bank Group’s FEEDHI are that it focuses mainly on the economic case of the FCM, and classifies digital health interventions based on how they utilise artificial intelligence (AI).

### 4.3. Limitations

Further sources relevant to the eHIAF for Africa may be available including unpublished sources and grey literature that have not been identified in this paper. Techniques such as a Delphi survey proposed by one respondent (C) may have helped to identify additional candidate attributes. The practical applicability of the eHIAF for Africa to the wide variety of eHealth initiatives in Africa will only be truly known as the framework is used. This will also answer a question posed by one respondent (A) about how the framework’s ‘robustness’ will be tested. Regarding completeness, the respondents suggested that the framework might be refined iteratively “*during implementation of the framework*”, to “*define a maturity framework*”, and to “*describe tools and approaches for implementing the framework*” (C). The framework could be further enhanced to include pre-appraisal questions “*to ensure time is not wasted on an economic appraisal on a non-viable initiative*” (H).

*Although* Table A4 *in the* Appendix A *guides the application of the eHIAF, there is still not a detailed step-by-step framework user guide that describes how to use the answers to each attribute question to decide whether or not to invest in an eHealth opportunity (G, H, J, K). A user guide could include an approach to attribute scoring (J), provide an “investment appraisal tool that is readily usable and configurable to… context” (G), and “help countries appreciate this tool” (K).*

### 4.4. Contribution of Prior Work

One respondent (H) noted that the two checklists used as the foundation for the eHIAF attributes combined two different perspectives, eHealth and economic appraisal, and asked for further clarity on how these checklists were incorporated. Earlier work determined that FCM “cases are applicable to African countries’ eHealth investment decisions” and produced the eHealth Investment Readiness Assessment Tool (eHIRAT) based on the FCM [62]. The eHIRAT allows a country to develop a profile of strengths and weaknesses in its eHealth environment to use to guide strengthening. Thereafter, a scoping review identified “eHealth investment appraisal approaches and tools that had been used in African countries, described their characteristics and made recommendations regarding African eHealth investment appraisal in the face of limited data and expertise” [47]. This resulted in the development of an extended FCM for digital health (FCM-DH) that, in combination with the economic perspectives of the economic appraisal checklist (the JBI checklist), together provided “the foundation of an African eHealth investment appraisal framework” [47] from which the eHIAF for Africa was subsequently developed [51].

## 5. Conclusions

The eHIAF for Africa is intended for use by officials working in resource-constrained settings who face the task of selecting the optimal eHealth investments despite any economics data and/or expertise limitations. Many of these officials are likely to be based in Africa working for governments, NGOs, and small companies interested in advancing eHealth. Based upon expert opinion, the eHIAF for Africa is considered ‘fit for purpose’. Indeed, the eleven respondents all indicated that they would use the eHIAF for Africa to evaluate future eHealth initiatives if it was strengthened based on the feedback they provided. Two respondents highlighted the potential for broader applicability of the framework beyond Africa.

The refinements proposed by the respondents were implemented including the addition of a flowchart, simpler attribute headings to make the framework easier to understand, and a revision of the descriptions of the attributes with additional detail added in a supporting narrative table to guide users to apply the framework. A further refinement was the change of Attribute 21 ‘Is there adequate connectivity?’ to the broader perspective ‘Is there an adequate ICT enabling environment?’ Only one comment can be regarded as having not been addressed: the suggestion to consider addressing emerging technologies such as AI and the Internet of Things. Only one respondent suggested this, and as noted above, it can be addressed indirectly using the eHIAF for Africa. The refined eHIAF for Africa is now ready for the development of user guides and practical tools, and thereafter the testing of its perceived usefulness to help African countries select the optimal eHealth initiatives that advance UHC.

## Figures and Tables

**Table 1:** Survey topics and question types.

Topic	Compulsory Closed Questions	Optional Open-Ended Responses
Respondent profile	7 Multiple choice	
Respondent’s perceptions of the availability of economics data and expertise in Africa	2 Likert scale	2
Respondent’s views of the framework development process	5 Likert scale; 1 Dichotomous	6
Respondent’s opinions regarding five qualities of the framework: structure, attributes, adequacy, usability, and uniqueness	30 Likert scale; 1 Dichotomous; 2 Multiple choice	31
Any further comments		1
Total	48	40

**Table 2:** Work environments and organisations of the respondents (Experts A to K).

	In Africa (LICs, LMICs, UMICs)	Outside Africa (LICs, LMICs)	Both within and outside Africa (LICs, LMICs)
Academia	1 (B)	1 (E)	
Local non-profit implementer	2 (C, D)	1 (G)	
Global NGO implementer	2 (F, A)	1 (K)	1 (H)
Global NGO normative guidance	1 (I)		1 (J)
	6	3	2

**Table 3:** Summary of the results of the compulsory Likert scale questions about the framework.

Aspect of the New Framework	Number of Questions (Total Number of Possible Responses)	Total Responses		
		Agree *	Unsure	Disagree **
Development process	5 (55)	50 (91%)	4 (7%)	1 (2%)
Structure	3 (33)	29 (88%)	2 (6%)	2 (6%)
Content	26 (286) ^†^	264 (92%)	18 (6%)	4 (1%)
Completeness	1 (11)	10 (91%)	0	1 (9%)
Total	35 (385)	353 (92%)	24 (6%)	8 (2%)

* ‘Mostly agree’ or ‘entirely agree’. ** ‘Mostly disagree’ or ‘entirely disagree’. ^†^ The sum of the % totals in this row is 99% due to a roundoff error when rounding to a whole number.

**Table 4:** Summary of the respondents’ opinions on whether each attribute is required (scores: Agree entirely = 5, Mostly agree = 4, Unsure = 3, Mostly disagree = 2, Disagree entirely = 1).

Attribute	Number of Responses for Each Score					Median Score
	5	4	3	2	1	
1. Is there a strategic fit between the eHealth initiative and the health strategy?	8	3				5
2. Is there a case for change?	8	3				5
3. Is there evidence of adequate stakeholder engagement in the appraisal process?	8	3				5
4. Does the appraisal include all issues of concern to users?	4	5	2			4
5. Are the results generalisable to the setting of interest?	5	4	2			4
6. Has an appropriate timescale been set?	6	5				5
7. Are all important and relevant costs and outcomes for each alternative identified?	5	4	1	1		4
8. Have appropriate assumptions and estimates been established?	6	4	1			5
9. Are costs and outcomes measured accurately and valued credibly?	5	5	1			4
10. Are costs and outcomes adjusted for differential timing (discount rate)?	4	6	1			4
11. Is there an analysis of options?	5	5	1			4
12. Is there an incremental analysis of costs and consequences for these options?	6	4	1			5
13. Is the risk exposure addressed and an adjustment made for optimism bias?	7	2	2			5
14. Were sensitivity analyses conducted to investigate uncertainty in estimates of cost or consequences?	4	5	1	1		4
15. Has clinical effectiveness been established?	8	3				5
16. Is affordability addressed?	7	4				5
17. Is there a sustainable business case?	9	2				5
18. Is there a practical plan for delivery?	8	2	1			5
19. Are clear delivery milestones provided?	7	3	1			5
20. Is change management addressed?	10	1				5
21. Is there adequate connectivity?	4	7				4
22. Is there a plan for building partnerships?	8	2		1		5
23. Is there a procurement plan?	6	5				5
Totals	148	87	15	3	0	
	235		15	3		

**Table 5:** Alignment between the five recommendations from a systematic review addressing the measurement of the value of the digital health initiatives and the eHIAF for Africa.

Recommendation Summary	How the Recommendation is Addressed in the eHIAF for Africa
Choose a comparator	Attribute 2 ‘case for change’ requires that a current standard of care comparator be identified and attributes 11 ‘options analysis’ and 12 ‘test for incremental shifts’ determine the incremental advantage of the proposed initiative compared to the comparator.
Have a multi-stakeholder perspective	Attribute 4 ‘user concerns’ ensures that sufficient issues of concern to users have been included and attribute 12 ‘test for incremental shifts’ quantifies incremental differences it delivers to beneficiaries.
Organisational impact should be protected	Attribute 19 ‘practicality’ addresses the arrangements needed for successful implementation including data governance and ensuring that data and knowledge generated by the initiative is accessible by health workers to be used for healthcare benefits, and attribute 20 ‘change management’ includes the need to confirm that the health care system is able to implement the changes required to realise the intended benefits.
Ensure multidimensional clinical, organisational, behavioural, and technical outcomes	The first 4 attributes help to clarify the value expectations that stakeholders have across multiple dimensions including clinical, organisational, behavioural, and technical; and attribute 5 ‘generalisability’ explores opportunities for further value enhancement by exploring the transferability of findings to other health settings and sectors with similar characteristics.
Address interoperability between data sources	Attribute 21 ‘infrastructure’ includes the need for interoperability to support data sharing.

## Data Availability

The datasets supporting the conclusions of this article are presented in the manuscript and can be further clarified with the corresponding author if required.

## Appendix A

**Table A1:** Summary of the structure of the proposed new eHIAF for Africa.

FCM Case	eHIAF Stages	eHIAF Appraisal Attributes *
Strategic	1. Establish a compact with key stakeholders	Is there a strategic fit between the eHealth initiative and the health strategy?
		2. Is there a case for change?
		3. Is there evidence of adequate stakeholder engagement in the appraisal process?
		4. Does the appraisal include all issues of concern to users?
		5. Are the results generalisable to the setting of interest?
Economic	2. Collect data	6. Has an appropriate timescale been set?
		7. Are all important and relevant costs and outcomes for each alternative identified?
		8. Have appropriate assumptions and estimates been established?
		9. Are costs and outcomes measured accurately and valued credibly?
		10. Are costs and outcomes adjusted for differential timing (discount rate)?
	3. Generate economic model	11. Is there an analysis of options?
		12. Is there an incremental analysis of costs and consequences for these options?
		13. Is the risk exposure addressed and an adjustment made for optimism bias?
		14. Were sensitivity analyses conducted to investigate uncertainty in estimates of cost or consequences?
		15. Has clinical effectiveness been established?
Financial	4. Establish affordability metrics	16. Is affordability addressed?
	5. Iterate to consider options and identify optimal investment choices	17. Is there a sustainable business case?
Management	6. Establish sustainable implementation	18. Is there a practical plan for delivery?
		19. Are clear delivery milestones provided?
		20. Is change management addressed?
		21. Is there adequate connectivity?
Commercial		22. Is there a plan for building partnerships?
		23. Is there a procurement plan?

Legend: FMC = Five Case Model, eHIAF = eHealth Investment Appraisal Framework. * The numbering of the eHIAF appraisal attributes indicates the order in which the attributes should proceed.

**Table A2:** List of the survey questions and question types.

Question Type	Likert	Multiple Choice	Dichotomous	Free Text
**Number of Questions**	37	9	2	40
**Part 1 of 6: Participant information (6 questions) and perceptions of the availability of economic data and expertise in Africa (2 questions)**				
1.1. What type of countries do you work in most? (Select all that apply from World Bank definitions: LIC, LMIC, UMIC, HIC)		×		
1.2. List up to five (5) countries that you have worked in most		×		
1.3. Which country do you work in most of the time? (Name one)		×		
1.4. What type of organisation do you work for? (Select one: Government, Private for-profit company, NGO or non-profit company implementing digital health solutions, NGO providing oversight and normative guidance, Academic institution, Other)		×		
1.5. What is your primary role? (Select one: Executive or senior manager responsible leading teams, programme manager responsible for managing digital health implementations, programme manager responsible for health programme implementations, technical expert responsible for aspects of building digital health solutions, Academic, Other)		×		
1.6.1 I have used cost effectiveness analysis to appraise an eHealth initiative. (Select one: Yes, no, I don’t know)		×		
1.6.2 I have used cost–benefit analysis to appraise an eHealth initiative. (Select one: Yes, no, I don’t know)		×		
1.7. I find that sufficient economic data are typically available in low- and middle-income countries (LMICs) to conduct eHealth investment appraisals	×			
1.8. I find that sufficient economic expertise is typically available in LMICs to conduct eHealth investment appraisals	×			
**Part 2 of 6: Impressions of the development of the new framework (6 questions)**				
2.1. The progression of the four steps is logical: (1) Identify required framework attributes, (2) Identify and review relevant frameworks, (3) Analyse available frameworks iteratively, (4) Develop new framework	×			×
2.2. The first of the four steps is appropriate: Identify required framework attributes from published checklists	×			×
2.3. The second of the four steps is appropriate: Select, review, and chart relevant frameworks using a scoping review	×			×
2.4. The third of the four steps is appropriate: Analyse the frameworks using deductive and inductive iterations	×			×
2.5. The fourth of the four steps is appropriate: If necessary (if an appropriate framework was not identified in Step 3) develop a new framework using the findings from the first three steps	×			×
2.6. Are you aware of any other framework (in addition to the five selected in the scoping review) that would provide useful information to improve the proposed new eHIAF? (Select Yes or No)			×	×
**Part 3 of 6: Impressions of the structure of the new framework (3 questions)**				
3.1 The structure is easy to understand	×			×
3.2 Aligning each ‘appraisal attribute’ with one of the ‘cases’ of the Five Case Model helps a user to understand the structure of the framework	×			×
3.3 Aligning each ‘appraisal attribute’ with one of the eHIAF stages helps a user to understand the structure of the framework	×			×
**Part 4 of 6: Impressions of the content of the new framework (26 questions)**				
4.1. Attribute 1 “Is there a strategic fit between the eHealth initiative and the health strategy?” is required	×			×
4.2. Attribute 2 “Is there a case for change?” is required	×			×
4.3. Attribute 3 “Is there evidence of adequate stakeholder engagement in the appraisal process?” is required	×			×
4.4. Attribute 4 “Does the appraisal include all issues of concern to users?” is required	×			×
4.5. Attribute 5 “Are the results generalisable to the setting of interest?” is required	×			×
4.6. Attribute 6 “Has an appropriate timescale been set?” is required	×			×
4.7. Attribute 7 “Are all important and relevant costs and outcomes for each alternative identified?” is required	×			×
4.8. Attribute 8 “Have appropriate assumptions and estimates been established?” is required	×			×
4.9. Attribute 9 “Are costs and outcomes measured accurately and valued credibly?” is required	×			×
4.10. Attribute 10 “Are costs and outcomes adjusted for differential timing (discount rate)?” is required	×			×
4.11. Attribute 11 “Is there an analysis of options?” is required	×			×
4.12. Attribute 12 “Is there an incremental analysis of costs and consequences for these options?” is required	×			×
4.13. Attribute 13 “Is the risk exposure addressed and an adjustment made for optimism bias?” is required	×			×
4.14. Attribute 14 “Were sensitivity analyses conducted to investigate uncertainty in estimates of cost or consequences?” is required	×			×
4.15. Attribute 15 “Has clinical effectiveness been established” is required	×			×
4.16. Attribute 16 “Is affordability addressed?” is required	×			×
4.17. Attribute 17 “Is there a sustainable business case?” is required	×			×
4.18. Attribute 18 “Is there a practical plan for delivery?” is required	×			×
4.19. Attribute 19 “Are clear delivery milestones provided?” is required	×			×
4.20. Attribute 20 “Is change management addressed?” is required	×			×
4.21. Attribute 21 “Is there adequate connectivity?” is required	×			×
4.22. Attribute 22 “Is there a plan for building partnerships?” is required	×			×
4.23. Attribute 23 “Is there a procurement plan?” is required	×			×
4.24. The order of the 23 attributes is appropriate	×			×
4.25. The proposed grouping of attributes into 6 stages is appropriate	×			×
4.26. The attribute descriptions are easy to understand	×			×
**Part 5 of 6: Impressions of completeness of the new framework (1 question)**				
5.1 The eHIAF addresses all important eHealth investment appraisal issues	×			×
**Part 6 of 6: Impressions of the utility of the new framework. Regarding whether you would use the proposed eHIAF, including any revisions from 5.1 (4 questions)**				
6.1. If applied effectively, the eHIAF (including any revisions you added under 5.1) will help to identify the optimal eHealth initiatives to be prioritised. (Select one: Agree, Disagree)			×	×
6.2. Would you use it to appraise any of the potential future eHealth initiatives that you become involved with? (Select one: No, Yes if strengthened based on my feedback in this questionnaire, Yes it is adequate as it is)		×		×
6.3. Would you use it to appraise any of your existing eHealth initiatives? (Select one: No, Yes if strengthened based on my feedback in this questionnaire, Yes it is adequate as it is)		×		×
6.5. If there are any additional comments you would like to make about the proposed eHIAF, please do so here.				×

**Table A3:** Amendments to the eHIAF for Africa including the expert who proposed the amendment in brackets where applicable, and an indication of how the framework was amended.

Affected Attributes	Comments Provided by Respondents	Refinements Applied to the Flowchart (Figure 1) and Narrative (Appendix A Table A4)
All	Add attribute names while retaining the attribute question (H).	Added attribute names.
All	Add a flowchart (K) Provide for a simple presentation of the framework that is backed up by more detailed guidance (J).	Flowchart added. Simplified the attribute questions where appropriate, and provided detailed descriptions of what the question means and how to apply it in a narrative table.
1. Strategic fit	Consider addressing compliance with local laws and regulations “*to ensure time is not wasted on an economic appraisal on a non-viable initiative*” (H).	Attribute 1 question refined to ‘Does the initiative align with the health strategy?’ Attribute 1 narrative expanded to include that the initiative should be compliant with local regulations.
3. Stakeholder engagement	“*Have ethical and equity issues been considered and planned for?*” (J). The framework should “*deal directly with governance*” (E); include governance in stakeholder engagement (J). “*Is there a data management plan?*” addressing issues such as security, privacy and consent (J); consider how to include “*confidentiality and security*” (D) Define ‘adequate’ (J, K).	Attribute 3 question simplified to “Are stakeholders adequately engaged?”. Attribute 3 narrative expanded to include (1) confirmation of which ethical and equity issues have been considered and how they will be addressed, (2) confirmation that there is a data governance plan, if appropriate, and (3) confirmation that issues such as security, confidentiality, privacy, and consent have been adequately addressed in stakeholder engagement activities. Attribute 19 narrative refined to include confirmation of the appropriateness of the governance arrangements The refinements clarified the definition of ‘adequate’.
4. User issues	Attribute question ‘Does the appraisal include all issues of concern to users?’ might work better if ‘all’ was changed (H); that ‘all’ changed to ‘most’ (C).	Attribute 4 question simplified to ‘Are issues of concern to users addressed’. The narrative for both attribute 3 and attribute 4 updated to include confirmation with stakeholders that these issues are adequately addressed.
5. Generalisability	Consider the term “*re-usability*” recognising that some outputs can “*enable or contribute to solutions for other sectors*” (I). Define ‘generalisable’ (B, C, E).	Attribute 5 question simplified to ‘Are the results generalisable to the setting?’. Attribute 5 narrative expanded to include that cross-sector usability should been considered and factored into design choices, and the refinements have clarified the definition of ‘generalisable’.
6. Time scale	Reinforce the need for continuous investment throughout the lifetime of a project and for large projects such as EMRs to have timescales of decades rather than years (J).	Attribute 6 narrative refined to reinforce the need for continuous investment throughout a project’s life cycle.
7. Costs and outcomes lists	Define ‘relevant’ (E).	Attribute 7 question simplified to ‘Are all relevant costs and outcomes identified?’ and the meaning of ‘relevant’ has been clarified in the narrative.
8. Assumptions and estimates lists	Define ‘appropriate’ (E).	The meaning of ‘appropriate’ has been clarified in the narrative.
9. Costs and outcome measurements	Recognising the “*work of the DHIF using the LiST and addressing morbidity avoided and lives saved estimates*” (K). Suggestion that “*certain outputs delivered as part of an eHealth initiative can also enable or contribute to solutions for other sectors*” (I).	Attribute 9 narrative refined to include (1) recognition of using estimate tools such as LiST, and (2) exploring synergies for cost and benefits sharing with other initiatives and other sectors.
10. Present value discounting	Define ‘discount rate’ (J).	A definition for ‘discount rate’ has been added to the narrative.
11. Options analysis	Expand “*how to reinforce the importance of comprehensive and adequate options analysis*” (K). Define ‘analysis of options’ (C, H).	Attributes 11 and 17 narratives refined to emphasise importance of options analysis. The definition of ‘analysis of options’ is clarified in the narrative.
12. Test for incremental shifts	Define ‘consequences’ (C).	Attribute 12 question simplified to ‘Is there an incremental analysis of options?’. ‘Consequences’ has been replaced with ‘outcomes’ for consistency.
13. Risk and bias adjustments	Define ‘risk’ and ‘optimism bias’ (H).	Attribute 13 question simplified to ‘Are risk exposure and optimism bias addressed?’. ‘Risk’ and ‘optimism bias’ have been explained in the narrative.
14. Sensitivity testing	No comments.	Attribute 14 question simplified to ‘Were sensitivity analyses conducted?’.
15. Clinical effectiveness testing	Extend clinical effectiveness “*beyond clinical to include public health benefit*” (C).	Attribute 15 narrative expanded to include that, wherever an initiative and its outcomes affect the health system level, this is appropriately explained and evidence provided.
16. Affordability	Include “*amortisation over the life-span of the digital health intervention*” when dealing with costs and outcomes (K).	Attribute 16 narrative refined to specify the inclusion of amortisation
18. Milestones	Consider the relationship between attribute 18 ‘Is there a practical plan for delivery?’ and attribute 19 ‘Are clear delivery milestones provided?’ (A, G).	The order of attributes 18 and 19 was switched and their narratives revised to clarify what each should address.
19. Practicality	Define ‘practical plan’ in attribute 18 (A).	The meaning of ‘practical plan’ was clarified.
20. Change management	Include “*political economy*” in the attribute dealing with change management (F). The framework should “*call out human resources as it does connectivity*” (H) including “*a clear human resource plan*” (J).	Attribute 20 narrative refined to include issues related to political economy. Human capacity added to the narratives of attributes 7 and 20.
21. Infrastructure	Include *‘IT infrastructure’* rather than singling out connectivity (C), addressing “*digital infrastructure more broadly*” (I) including issues such as “*infrastructure availability*” (A) and expand the attribute to include “*power availability and hosting services*” and “*the policy enabling context—including data security, privacy, confidentiality, sharing, and exchange*” to create “*an adequate ICT enabling environment*” (K).	Attribute 21 renamed to ‘Is there an adequate ICT enabling environment?’ and refined the narrative to address the respondents’ suggestions.
22. Partnerships	Expand the question on partnerships to “*include all relevant sectors*” (C).	Attribute 22 narrative refined to emphasise that relevant cross-sector partnerships should be considered.
23. Procurement	Procurement plans should include transition to a “*sustainability model*” and that sustainability could be separated into a “*a group of its own… due to its importance*” (C).	Attribute 23 narrative refined to include that procurement plans should address sustainability by clarifying how close-out will be handled and sustainability addressed.

**Table A4:** List of the attributes, definitions, and descriptions of how each attribute should be used in an eHealth investment appraisal.

Attribute Name	Attribute Question	Requirements for Implementing Each Attribute
Strategic Case		
1. Strategic fit	Does the initiative align with the health strategy?	Clear alignment exists between the initiative and the health policy context; the objective is clearly stated and reflects the main stakeholders’ perspectives; there is an appropriate mix of initiatives to offer the best fit to health strategies; and the initiative is compliant with local regulations.
2. Case for change	Is there a case for change?	There is a clear and concise statement of the required service outputs and requirements and how they compare to a standard of care, with explicit, measurable, time-bound investment objectives and indication of the current standard or ‘comparator’ that will be improved upon.
3. Stakeholder engagement	Are stakeholders adequately engaged?	Stakeholders have been adequately engaged. That is, they have been consulted to: Confirm the case for change and strategic context; Identify users’ expectations regarding required features across clinical, organisational, behavioural, and technical dimensions, including confirmation by intended users that sufficient issues of concern to them are included in the appraisal; Re-design work practices for optimal fit; Ensure that all important and relevant costs and outcomes for each alternative are identified; Capture user perceptions, achieve “buy-in” and establish consensus when analysing options; Confirm governance arrangements for the implementation of the DHI, including which ethical and equity issues been considered and how they will be addressed, with specific clarifications regarding security, confidentiality, privacy and consent and a data governance plan if appropriate.
4. User issues	Are issues of concern to users addressed?	The initiative addresses all the questions decision-makers would ask when deciding about whether or not to proceed with the initiative by addressing stakeholders’ improvement expectations (such as demonstrating value for money to encourage use) aligned with their perceptions, as confirmed in stakeholder consultations
5. Generalisability	Are the results generalisable to the setting?	The implementation setting is adequately described and the opportunity for transferability of findings to other settings and sectors with similar characteristics has been considered explicitly and factored into design choices.
**Economic Case**		
6. Timescale	Has an appropriate timescale been set?	The timescale describes and justifies the entire life cycle of the initiative from conceptualisation, through approval and implementation, through any reinvestment phases and on to obsolescence and decommissioning, recognising that large projects (such as EHRs) have timescales of decades rather than years.
7. Costs and outcomes lists	Are all relevant costs and outcomes identified?	There is a comprehensive list of all cost and outcomes for each alternative that are relevant (which means those sufficient to address the objectives of the project), reinforcing the need for continuous investment throughout the lifetime of a project including human resources and all the intended changes are compiled into a benefits register.
8. Assumptions and estimates lists	Have assumptions and estimates been established?	All appropriate and relevant assumptions have been recorded, and the costs and outcome estimates have been specified with accompanying ranges.
9. Costs and outcomes measurements	Are they (costs and outcomes) measured accurately and valued credibly?	Selected measurement methods are justified, and limitations are discussed, recognising that in economic evaluations it is often difficult to measure the costs and outcomes accurately, and hence this quality criterion may be difficult to achieve. A persuasive argument is provided for the pricing method used to value the costs and benefits such as using the LiST and addressing morbidity avoided and lives saved estimates described in DHIF. Synergies with other initiatives and other sectors are explored to identify opportunities for cost sharing (to reduce the total cost carried by the eHealth initiative) and benefits sharing (to maximise the benefits).
10. Present value discounting	Are they (costs and outcomes) adjusted for differential timing?	A discount rate (the interest rate or rate of return used to discount future cash flows back to their present value) is provided, with sufficient justification, aligned with the timescale provided in Step 6, and applied to the costs and outcomes.
11. Options analysis	Is there an analysis of options?	Because comprehensive options analysis is critical to determining the optimal business case, a clear description of the initiative(s) and comparator(s) is provided to identify options that offer the best affordable value for money and find the optimal fit for the organisation’s business needs. Analyses of each of the options are provided to and reviewed with the key stakeholders including the users to consider which options provide the best net-benefit and strategic fit.
12. Test for incremental shifts	Is there incremental analysis of the options?	A measure is reported that shows the change in the costs and outcomes for the initiative and a comparator for a marginal shift in resources from the comparator to the intervention.
13. Risk and bias adjustments	Are risk exposure and optimism bias addressed?	Key risks (issues that increase uncertainty or create potential for increased costs and fewer benefits) have been entered into a risk register, analysed and costed, for use in sensitivity testing. Optimism bias (a situation in which the analyst believes that there is less risk associated with an initiative than the average population would believe) has been examined and applied to the initial estimates. Optimism bias adjusted for risk is reflected in the level of certainty shown by the costs and outcomes value ranges. There is a description of how the risks, uncertainties, and optimism bias have been estimated.
14. Sensitivity testing	Were sensitivity analyses conducted?	Sensitivity analyses were conducted to investigate uncertainty in the estimates of cost or consequences, testing results are presented to confirm the robustness of findings, describing how findings vary with changes in key variables such as relative prices and intervention estimates. Early estimates of key benefits and key risks have been entered into the benefits and risks registers, respectively, and been used in sensitivity analysis.
15. Clinical effectiveness testing	Has clinical effectiveness been established?	The evidence used to derive a clinical effectiveness estimate (which may be derived from another initiative), the level of this evidence, and how the estimate was derived are recorded. Where the initiative and its outcomes affect the health system level, rather than the individual clinical level, this is appropriately explained, and sufficient evidence provided.
**Financial case**		
16. Affordability	Is the initiative affordable?	The initiative’s potential and the whole life costs (capital and revenue) over the entire life span of the initiative and sources of funding are clearly identified, agreed among stakeholders, and affordable. The costs of monitoring and evaluating, and procurement are included. All of the indicative financial cost ranges, sources, and assumptions have been updated with the best estimates available. The sum of residual optimism bias and residual risk have been revisited as a basis for estimation of the contingent cost liability. Contingent cost liabilities have been addressed, informed by a likelihood valuation. Options for amortisation over the lifespan of the initiative have been considered.
17. Iterations	Is there a sustainable business case?	Sufficient iterations have been performed to refine the model to establish the optimal link between socioeconomic returns and affordability, with the impacts on the balance sheets and cash flow of participating organisations over the time reviewed and decision-makers in agreement with the business case for the life cycle of the investment.
**Management case**		
18. Milestones	Are clear delivery milestones provided?	There are clear milestones (deliverables and dates) for initiative outputs including key contractual and delivery arrangements relating to these dates.
19. Practicality	Is there a practical plan for delivery?	A practical plan clarifies the operational plans and resources needed for the successful implementation of the initiative including working arrangements for governance, monitoring and reporting, assurance and post evaluation, and ensuring that the data and knowledge generated by the initiative is accessible by health workers to be used for healthcare benefits. The plan also allocates risks to the organisations best placed to monitor and manage them and has contingency plans in place to address their occurrence.
20. Change management	Is change management addressed?	A plan is in place to support stakeholders, particularly users, through the changes that need to be made to ensure success of the initiative including human resource changes and redeployments and the associated capacity development needs, confirming that the health system is able to implement the changes required to realise the intended benefits.
21. Infrastructure	Is there an adequate ICT enabling environment?	There is a plan for each milestone of the initiative to ensure that sufficient ICT infrastructure (including electricity, software, hardware, connectivity) will be in place for the initiative’s success and that plausibly addresses all of the unique local setting challenges; interoperability is in place to support appropriate data sharing; adequate clarifications are provided regarding how security and privacy will be protected.
**Commercial case**		
22. Partnerships	Is there a plan for building partnerships?	Due diligence has been undertaken to identify any capacity, resilience, and capability gaps and to recruit partners or contractors to fill them. Sufficient evidence is provided listing all partnerships or other contracts that will be required, confirming that all required deals are likely to be achieved. All envisaged deals have been summarised together with details of the service outputs, timescales, risk apportionment, payment mechanisms, and accountancy treatment including the accounting treatment of underpinning assets. There are key contractual and delivery milestones and dates. The potential for risk transfer among partners has been addressed including how risk will be tied down in payment arrangements. There are robust and enforceable commercial contracts with appropriate contractual clauses.
23. Procurement	Is there a procurement plan?	There is a clear understanding of the procurement approach. There is a draft advertisement for competitive procurement. Contract lengths have been stipulated, together with any required breakpoints. A joint approach has been agreed with any other affected entities and arrangements are in place to manage that. Investment objectives reflect any adjustments made as a result of procurement. Procurement plans address sustainability by clarifying how close-out will be handled including transition to a ‘sustainability model’ where appropriate.
